# Awareness About the Protection of Children From Sexual Offences Act Among Healthcare Professionals and Their Role in the Care of Child Sexual Abuse Victims

**DOI:** 10.7759/cureus.80598

**Published:** 2025-03-14

**Authors:** Sameer S Patra, Sindhu Sankaran, Amit Satapathy, Rashmi R Das, Joseph John

**Affiliations:** 1 Pediatric Medicine, All India Institute of Medical Sciences, Bhubaneswar, Bhubaneswar, IND; 2 General Surgery, All India Institute of Medical Sciences, Bhubaneswar, Bhubaneswar, IND; 3 Pediatrics, All India Institute of Medical Sciences, Bhubaneswar, Bhubaneswar, IND; 4 Pediatrics and Child Health, All India Institute of Medical Sciences, Bhubaneswar, Bhubaneswar, IND

**Keywords:** attitude, child, child abuse, healthcare providers, knowledge, practice, sex offence

## Abstract

Introduction: Child sexual abuse (CSA) is prevalent worldwide. Many medical professionals feel uncomfortable or lack the skills required to manage cases of sexual abuse.

Objectives: To assess the extent of knowledge, attitude, and practice (KAP) regarding the Protection of Children From Sexual Offences (POCSO) Act 2012 amongst healthcare professionals.

Methods: This cross-sectional study was conducted in a tertiary care teaching hospital in Eastern India. After pre-testing, medical professionals were administered a questionnaire containing sections on knowledge, attitude, and practice, including healthcare workers' role in managing CSA victims. Statistical analysis was done using STATA software.

Results: Out of a total of 403 medical professionals who participated, in the knowledge category, >60% did not know the age for obtaining consent from the child, the organization interested in monitoring and implementing the act, the conditions when compensation should be awarded to the child, and the punishment if the medical professional fails to report the case under the act. In the practice category, >90% did not do the following: provide first aid to the child before asking questions related to CSA, estimate the age of the child, hand over the evidence collected after examination to the parents or caregivers, follow up with the child, or provide a medical certificate to the parents or caregivers when coming across a child with CSA. The attitude parameters were satisfactory.

Conclusions: The present study showed that the knowledge and practice of the POCSO Act amongst medical professionals was highly unsatisfactory. Periodic training of medical professionals and strengthening the legislature is the need of the hour.

## Introduction

Child sexual abuse (CSA), as defined by the World Health Organization (WHO) Consultation on Child Abuse Prevention 1999, is the involvement of a child in a sexual activity that they do not fully comprehend, is unable to give informed consent to, or for which the child is not developmentally prepared and cannot give consent, or that violates the laws or social taboos of society [1]. CSA is a unique entity and cannot be dealt with in the same way as adult sexual abuse victims. Some characteristics of CSA are manipulation of trust rather than using physical force to hide the abuse; the abuser is generally a known person to the child. Intra-familial abuse accounts for about one-third of all CSA cases; it is usually chronic, with repeated episodes happening over weeks to years and the invasive nature escalating over time [2].

CSA is a pressing issue that is increasing in magnitude throughout the world. The prevalence of sexual abuse in the world is estimated to be 7% among girls and 7.9% among boys below the age of 18 [3]. India accounts for the world’s largest population of CSA victims. Many cases fail to be reported due to various socio-cultural factors and taboos, and children are not developmentally sound enough to realize that they are being abused and fear the abuser, who might be a known person or close family member.

CSA has profound adverse consequences on the child’s health, including failure of proper growth and development, poor mental and social health outcomes, relational challenges, substance abuse, and even increased risk of becoming perpetrators of sexual abuse as adults [4-6].

Recognizing the need in India, the Prevention and Protection of Children from Sexual Offences (POCSO) Act 2012 emerged as a comprehensive law to provide for the protection of children from sexual assault, sexual harassment, and pornography while safeguarding and providing child-friendly mechanisms along every step of the judicial process [7]. It also clearly states the role of medical professionals, including proper examination, which causes as little distress to the child as possible.

It is extremely crucial that all healthcare professionals are well aware of the POCSO Act and its guidelines about the responsibilities of medical personnel. There are few studies regarding the awareness of the management of CSA among healthcare workers and none regarding the POCSO Act [8,9]. This study was conducted to determine the extent of healthcare providers' knowledge about the POCSO Act, the management of CSA, and the attitude of medical professionals toward CSA in a tertiary care center.

## Materials and methods

This cross-sectional, questionnaire-based (Appendix) study was conducted amongst medical professionals in a tertiary care teaching institute in Eastern India. The study was conducted over four months in the Department of Pediatrics from September 2019 to December 2019. The medical professional included all doctors, including junior residents, senior residents, faculties, medical students, nursing students, and nursing staff. First-year medical and nursing undergraduate students (as they are newly enrolled in the course) and those participating in another study at the same time were excluded.

After ethical clearance, first, a pretest of the questionnaire with a sample size of 30 was done. Each questionnaire consisted of 31 questions: 15 were related to knowledge, nine were related to attitude, and seven were related to practice. All were multiple-choice questions (MCQs). After making the required modifications to the questionnaire, medical professionals were approached with the self-administrable questionnaire containing various sections on knowledge, attitude, and practice (KAP) regarding the various principles of the POCSO Act 2012, including the role of healthcare workers in the management of CSA victims. The study's purpose and procedure for filling out the questionnaire were explained to the participants.

Sample size calculation

Considering that no prior study had been done before, taking 50% as the prevalence of CSA with an alpha level of 5%, the total sample size was calculated to be 400.

Statistical analysis

As this was a qualitative study based on a questionnaire, descriptive statistics were used to present the data. Statistical analysis was conducted using STATA software.

## Results

A total of 403 medical professionals participated. There were 227 males (56.3%) and 176 females (43.7%). All the participants responded to all the questions. The characteristics of medical professionals who participated in the study have been described in Table 1.

**Table 1:** Characteristics of the healthcare professionals (total of 403 participants).

Characteristics		Number (%)
Gender	Male	227 (56.3)
	Female	176 (43.7)
Age (Mean±SD)	Male	24.64 ±5.9
	Female	23.47± 4.8
Designation	Undergraduate students	247 (61.3)
	Junior resident	45 (11.2)
	Senior resident	61 (15.1)
	Nursing student	38 (9.4)
	Nursing officer	12 (3.0)
Departments	Pediatrics	27 (6.7)
	Obstetrics & Gynecology	18 (4.5)
	Forensic Medicine	6 (1.5)
	Psychiatry	8 (2.0)
	Others	344 (85.3)

**Figure 1:**
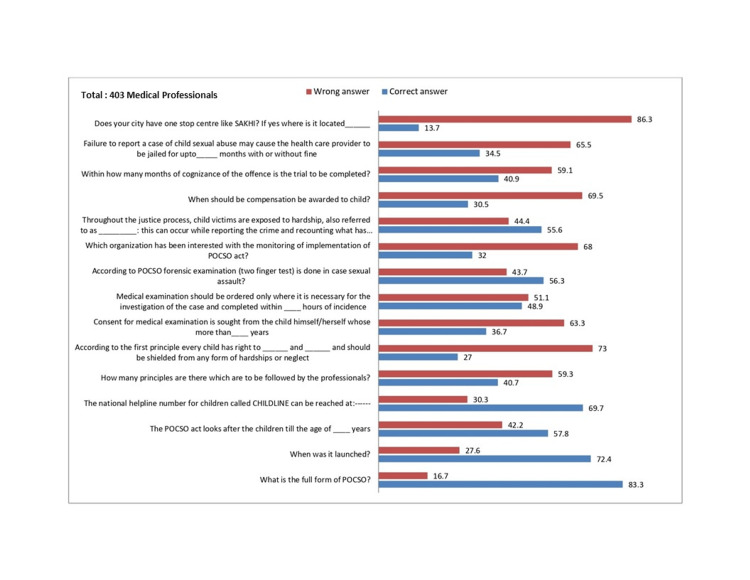
Knowledge about the POCSO act. The numbers on the top of the bars represent the % of responses (right). The questions are related to knowledge mentioned on the left (total of 403 participants).

Around 61% of the participants were undergraduate students, and 26% were residents (junior and senior). Approximately 83% of the participants had some knowledge about child abuse and the POCSO Act. In the knowledge category, >60% did not know about the following: the age for consent from the child, the organization interested in monitoring and implementing the act, when compensation should be awarded to the child, and the punishment if the medical professional fails to report the case under the act as shown in Figure 1.

**Figure 2:**
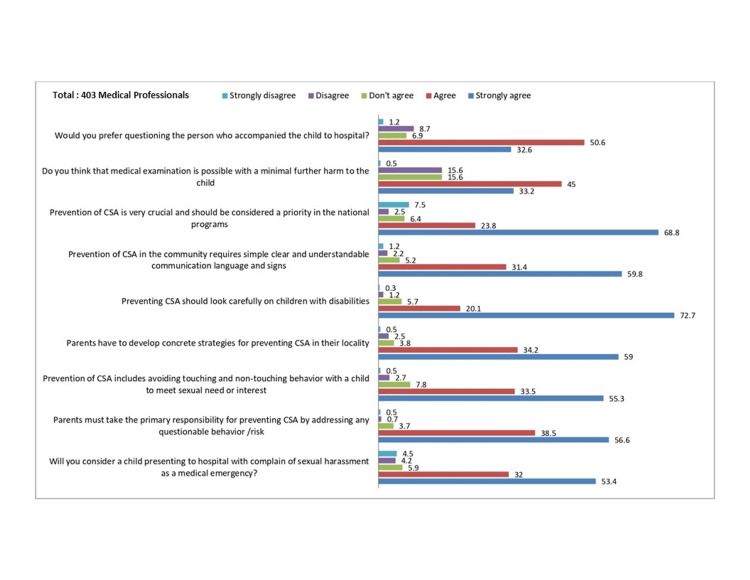
Attitude about the POCSO act. The numbers on the top of the bars represent the % of responses (right). The questions related to attitude are mentioned on the left (total of 403 participants).

The attitude parameters were satisfactory, as shown in Figure 2.

**Figure 3:**
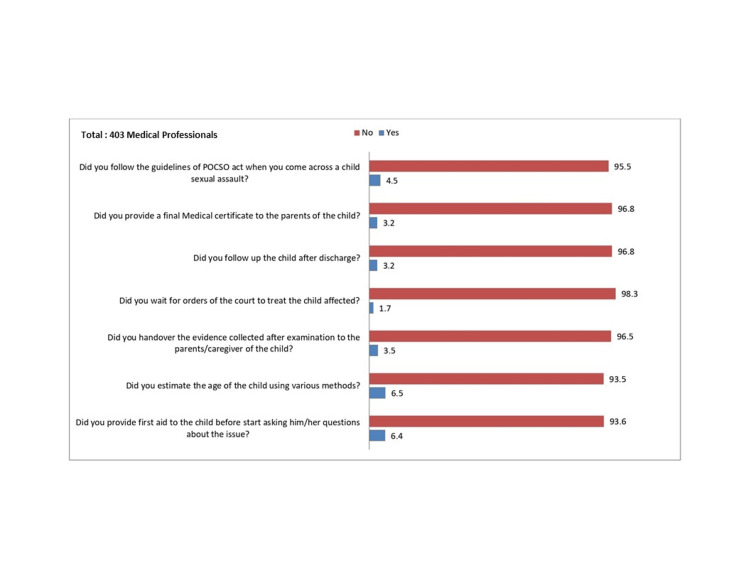
Practice of the POCSO act. The numbers on the top of the bars represent the % of responses (right). The questions related to practice are mentioned on the left (total of 403 participants).

In the practice category, >90% did not do the following: provide first aid to the child before asking questions related to CSA, estimate the age of the child, hand over the evidence collected after examination to the parents or caregivers, follow up with the child, or provide a medical certificate to the parents or caregivers when coming across a child with CSA, as shown in Figure 3.

## Discussion

CSA encompasses many types of sexual maltreatment of children, including penetrative or non-penetrative sexual intercourse, sexual harassment, sexual exploitation of children for commercial purposes, sex tourism, and pornography [10]. A report by UNICEF tells us that CSA is a global and widespread phenomenon where approximately 4.5% of girls in India between the ages of 15 and 19 have been exposed to sexual violence [11]. These numbers are just a minor reflection of the true numbers, as many cases go unnoticed or unreported due to diverse socio-cultural factors. A survey amongst adolescents in Kerala, India, reported an astounding 36% and 35% of boys and girls who had experienced sexual abuse in their lives [12]. The UNICEF and Indian Medical Association joined hands in 2015 to combat CSA and came up with a teaching manual and 10 key messages to be distributed to more than 2.5 lakh doctors across India regarding the management of CSA as a medical emergency and treatment to be provided free of cost by both government and private hospitals [11].

In the present study, 83.3% of the participants had knowledge about child abuse and the POCSO Act. Whereas in a study done at a tertiary care teaching institute in Chandigarh, this figure was 81% [13]. In a study done in Ahmednagar, Maharashtra, this figure was 72.6% [14]. However, in a study from Chennai, only 45% of the participants were aware of the exact features of the POCSO Act [15]. The discrepancies noted amongst the studies may be related to the participant composition as well as sensitization about the POCSO Act during the course of teaching and training. In the present study, 26% were junior and senior residents, whereas, in the study from Chandigarh, 95% were junior and senior residents (this high number is because it is a post-graduate institute) [13]. The study from Ahmednagar consisted of medical practitioners, whereas that from Chennai consisted of students and interns. Regarding awareness about the age of children up to 18 years included in the act, 57% were correct in the present study and the study from Chennai, but in the Ahmednagar study, it was only 34%. The proportion of participants being aware of the helpline number (1098) and the punishment to be awarded in case of failure to report a case of CSA was nearly the same in the present study and the studies from Chandigarh and Ahmednagar. Awareness regarding the availability of a one-stop center at a respondent’s hospital was present in 70% of the Chandigarh study, in contrast to only 14% in the present study.

In the practice category, the present study showed a very poor response with >90% not adhering to most practice parameters outlined in the POCSO Act 2012. This was in contrast to other studies.

Though the study is unique of its kind as it evaluated all the parameters of the POCSO Act 2012 through a large (403 participants) questionnaire-based survey, it has certain important limitations. First, the risk factors of CSA, which may be useful to inform the government and policymakers, were not evaluated. Second, the findings still may not be generalizable as the proportions of participants in different subgroups were not uniform (undergraduate students form the majority of the respondents).

## Conclusions

The present study showed that the knowledge and practice of the POCSO Act among medical professionals were highly unsatisfactory.

## Appendices

Participant ID:

Age:

Gender:

Working as: Medical student/ JR/ SR/ Professor/ nursing student/ Nursing officer

Department:

Knowledge

1. What is the full form of POCSO?

A. Prevention Of Children against Sexual Offences

B. Protection Of Children from Sexual Offences

C. Persecution Of Children Sexual Offenders

D. Protection of Children against Sexual Offenders

2. When was it launched?

A. 2010 B. 2011 C. 2012 D. 2013

3. The POCSO act looks after the children till the age of ____ years

A. 12 B. 14 C. 16 D. 18

4. The national helpline number for children called CHILDLINE can be reached at:

A. 1098 B. 104 C. 101 D. 100

5. How many principles are there which are to be followed by the professionals?

A. 10 B. 11 C. 12 D.13

6. According to the first principle every child has the right to ______ and ______ and should be shielded from any form of hardships or neglect.

A. Life, survival B. live, prosper C. learn, prosper D. safety, growth

7. Consent for medical examination is sought from the child himself/herself who is more than____ years old.

A. 12 B. 14 C. 16 D.18

8. Medical examination should be ordered only where it is necessary for the investigation of the case and completed within ____ hours of incidence. 

A. 24 B.48 C.72 D.96

9. According to POCSO forensic examination (two-finger test) is done in case of sexual assault.

A. Yes B. No

10. Which organization has been entrusted with the monitoring of the implementation of the POCSO Act?

A. National commission for protection of child rights B. Juvenile justice court C. Ministry of women and child development D. National commission for women and child safety 

11. Throughout the justice process, child victims are exposed to hardship, also referred to as _________: this can occur while reporting the crime and recounting what has happened while awaiting trial and while testifying in court.

 A. Primary Victimization B. Secondary Victimization C. Tertiary Victimization D. Victimization

12. When should compensation be awarded to a child?

A. During the trial B. End of trial C. During and end of trial D. At any time

13. Within how many months of cognizance of the offense is the trial to be completed?

A. 3 months B. 6 months C. 9 months D. 12 months

14. Failure to report a case of child sexual abuse may cause the healthcare provider to be jailed for up to_____ months with or without a fine. 

A. 3 months B. 6 months C. 9 months D. 12 months

15. Does your city have a one-stop center like SAKHI? If yes where is it located ______Capital Hospital, Bhubaneswar

Attitude

Score the questions 16 to 25 On a scale of 1 to 5 where

1. Strongly Agree 2. Agree 3. Don't know 4. Disagree 5. Strongly disagree

16. Will you consider a child presenting to the hospital with a complaint of sexual harassment as a medical emergency?

­­­­­­­_________________

17. Parents must take the primary responsibility for preventing CSA by addressing any questionable behavior /risk

__________________

18. Prevention of CSA includes avoiding touching and non-touching behavior with a child to meet sexual needs or interests.

___________________

19. Parents have to develop concrete strategies for preventing CSA in their locality

___________________

20. Preventing CSA should look carefully at children with disabilities

___________________

21. Prevention of CSA in the community requires simple clear and understandable communication language and signs

___________________

22. Prevention of CSA is very crucial and should be considered a priority in the national programs

___________________

23. Will you consider a child presenting to the hospital with a complaint of sexual harassment as a medical emergency?

___________________

24. Do you think that a medical examination is possible with minimal further harm to the child

___________________

25. Would you prefer to question the person who accompanied the child to the hospital?

_____________________ 

Practice

26. Did you provide first aid to the child before start asking him/her questions about the issue? 

A. Yes B. No

27. Did you estimate the age of the child using various methods?

A. Yes B. No. If Yes, How__________?

28. Did you hand over the evidence collected after examination to the parents/caregiver of the child? 

A. Yes B. No

29. Did you wait for orders of the court to treat the child affected?

A. Yes B. No

30. Did you follow-up with the child after discharge? 

A. Yes B. No

31. Did you provide a final Medical certificate to the parents of the child?

A. Yes B. No

32. Did you follow the guidelines of the POCSO Act when you come across a child sexual assault? 

A. Yes B. No

## References

[1] World Health Organization, Geneva. Geneva. (2025). World Health Organization: Report of the consultation on child abuse prevention. World Health Organization.

[2] World Health Organisation, Geneva Geneva (2025). World Health Organization: Guidelines for medico-legal care for victims of sexual violence. https://iris.who.int/bitstream/handle/10665/42788/924154628X.pdf?sequence=1.

[3] Wihbey J (2025). The Journalist's Resource: Global prevalence of child sexual abuse. https://journalistsresource.org/criminal-justice/global-prevalence-child-sexual-abuse/.

[4] Choudhry V, Dayal R, Pillai D (2018). Child sexual abuse in India: a systematic review. PLoS One.

[5] Putnam FW (2003). Ten-year research update review: child sexual abuse. J Am Acad Child Adolesc Psychiatry.

[6] Sowmya BT, Seshadri SP, Srinath S (2016). Clinical characteristics of children presenting with history of sexual abuse to a tertiary care centre in India. Asian J Psychiatr.

[7] (2025). India Code: The protection of children from sexual offences act 2012. https://www.indiacode.nic.in/bitstream/123456789/2079/1/AA2012-32.pdf.

[8] Deshpande A, Macwan C, Poonacha KS (2015). Knowledge and attitude in regards to physical child abuse amongst medical and dental residents of central Gujarat: a cross-sectional survey. J Indian Soc Pedod Prev Dent.

[9] Blakeley J, Ribeiro V (1997). Community health and pediatric nurses' knowledge, attitudes, and behaviors regarding child sexual abuse. Public Health Nurs.

[10] Seth R, Srivastava RN (2017). Child sexual abuse: management and prevention, and protection of children from sexual offences (POCSO) act. Indian Pediatr.

[11] (2025). UNICEF: Strategy for ending violence against children. https://www.unicef.org/india/media/4151/file/End%20Violence%20Against%20Children%20Strategy%20India.pdf.

[12] Krishnakumar P, Satheesan K, Geeta MG, Sureshkumar K (2014). Prevalence and spectrum of sexual abuse among adolescents in Kerala, South India. Indian J Pediatr.

[13] Singh R, Koushal V, Bharti B (2022). A descriptive study on child sexual abuse act in India. J Family Med Prim Care.

[14] Kadu S, Shinde A, Mhaske SN (2021). Assessment of knowledge and attitude about POCSO act amongst medical practitioners. Ind Jr Med Pat.

[15] Satish S, Shruthi P (2021). Awareness about acts and amendments in bills related to sexual offences amongst medical students and interns in a private medical college and hospital at Chennai. Ind Jr Med Pat.

